# Developing sustainable service user involvement practices in mental health services in Sweden: the “Userinvolve” research program protocol

**DOI:** 10.3389/fpsyt.2023.1282700

**Published:** 2023-10-12

**Authors:** Urban Markström, Hilda Näslund, Ulla-Karin Schön, David Rosenberg, Ulrika Bejerholm, Anneli Gustavsson, Mårten Jansson, Elisabeth Argentzell, Katarina Grim, Patrik Engdahl, Faten Nouf, Sara Lilliehorn, Petra Svedberg

**Affiliations:** ^1^Department of Social Work, Umeå University, Umeå, Sweden; ^2^Department of Social Work, Stockholm University, Stockholm, Sweden; ^3^Department of Health Sciences, Lund University, Lund, Sweden; ^4^The Swedish Partnership for Mental Health, NSPH, Stockholm, Sweden; ^5^Department of Social and Psychological Studies, Karlstad University, Karlstad, Sweden; ^6^School of Health and Welfare, Halmstad University, Halmstad, Sweden

**Keywords:** mental health, co-production, service users, involvement, protocol, research program

## Abstract

**Background:**

The purpose of this paper is to outline the protocol for the research program “UserInvolve,” with the aim of developing sustainable, service user involvement practices in mental health services in Sweden.

**Methods:**

This protocol outlines the knowledge gap and aim of the UserInvolve-program. It further provides an overview of the research infrastructure, with specific focus on the organization and management of the program as well as the design of the six underlying research projects. These six research projects form the core of the UserInvolve-program and will be carried out during a six-year period (2022–2027). The projects are focused on examining articulations of experiential knowledge in user collectives, on four specific user involvement interventions (shared decision-making, peer support, user-focused monitoring, and systemic involvement methods) and on developing theory and method on co-production in mental health research and practice.

**Results or conclusion:**

The knowledge gained through the co-production approach will be disseminated throughout the program years, targeting service users, welfare actors and the research community. Based on these research activities, our impact goals relate to strengthening the legitimacy of and methods for co-production in the mental health research and practice field.

## 1. Introduction

Including the voices and knowledge of the service user (hereafter abbreviated to SU) is core to the delivery and quality development of mental health services and is recognized as an essential component both in Sweden and internationally (1–3). In Sweden, Government commissions of inquiry have highlighted SU involvement as a prioritized concern (4, 5) and there is a growing emphasis on specific methods and interventions to increase SU involvement (6, 7). However, involvement strategies are often implemented sporadically, and a lack of sustainable implementation has been highlighted (8). At the same time, the key role of SUs experiential knowledge as an essential ingredient in evidence-based practice (EBP) is increasingly recognized. In conjunction with professional and research-based knowledge, experiential knowledge constitutes one of the three components in EBP (9). The significance of integrating the SUs values, preferences and knowledge is also central to the framework of person-centred care (PCC) (10) promoted in strategic documents and policy (11, 12). From a personal recovery perspective, the importance of services being oriented towards the values and knowledge of SUs, is further emphasised (13, 14).

Close collaborations between public sector and SU movement actors distinguishes the Swedish context for SU involvement (15). In the mental health sector, SU organizations have since the 1990s been involved in government commissions of inquiry and national projects (16). These organizations today assume a key role in welfare system democratization and in the quality development of mental health services. Further, SU organizations drive the development of SU involvement methods in Sweden. Parallel with rather substantial changes regarding SU involvement in the field of mental health services, there is a trend in parts of the research community to emphasise strategies for co-production, meaning that researchers, practitioners, and the public work together, with the ambition to share power and responsibility throughout the research process. Guiding principles that are stressed often concern building relationships, sharing of power, reciprocity, and embracing diverse perspectives and skills (17). As with the issue of SU involvement in mental health services, attempts to co-produce research can be challenged by practical circumstances, including unequally distributed resources, limitations in arenas for participation for involved actors, and risks for both tokenism and co-optation (18). Incorporating user knowledge is acknowledged as a vital element in enhancing the provision and quality advancement of mental health care and social support, both within Sweden and on a global scale (6, 19, 20). Recent research, however, suggests that although there is a positive discourse about involving SUs, there are still challenges when it comes to legitimizing user knowledge in practical application (8). User participation often is reduced to tokenistic gestures, where SUs are not seen as equal partners in collaborative knowledge-sharing processes (21). Frequently, SUs raise concerns about their dependency on professionals and not being recognized as capable and trustworthy collaborators in knowledge processes (8, 22). In health research, experiential knowledge remains commonly regarded as anecdotal, while the significance of expert knowledge is amplified (23). While imbalances in knowledge validation and power are recognized as obstacles to participation in various care and support fields, studies have shown that in mental health settings, issues of disempowerment, stigma, and coercion can exacerbate these barriers (24, 25).

To address the knowledge gaps about strategies for involving SUs and co-producing in mental health research and practice, a research program is proposed with the aim of developing co-produced, cross-disciplinary, and practice-relevant research on SU involvement. This is a 6-year program, funded in May 2021 by FORTE (Swedish Research Council for Health, Working Life and Welfare) [grant number: 2021–01427], with the explicit ambition to promote a transformative change of mental health services through a co-production approach (26). This protocol provides a description of UserInvolve, with a specific focus on the organization and management of the program, as well as the design of the six underlying research projects, that together, yet from different perspectives, contribute to fulfill the aim of the program. Further, this paper illustrates the program’s aims and objectives, overarching theoretical perspectives and methods as well as the overall program organization and project-level activities. Finally, a time plan, including the impact goals and ethical considerations and dissemination strategies is presented.

This paper provides an outline for a 6-year research program focused on the issue of SU involvement in mental health services in Sweden. Special attention is directed to community mental health services provided by the local municipalities. The program aims to examine strategies for SU involvement at the individual-, organizational- and system level, and develop sustainable SU involvement through co-production processes involving the research community, welfare actors and SU organizations. The specific aims are to:

- Explore how experiential knowledge can be articulated, and how it can be legitimized within mental health practices.
- Examine barriers and success factors in the implementation of SU involvement methods in mental health practice.
- Examine and develop co-production methods for integrating SU involvement in research and mental health practice.

## 2. Theoretical starting points

The theoretical starting point of this research program relates to co-production, knowledge generation and more specifically, the power relations in the creation of knowledge and in SU involvement practices in general. Communities of Practice, CoP, is a social theory based on collective processes (27, 28). Knowledge is understood as something that arises in interpersonal communities where the knowledge process itself is in focus (29). A community of practice (CoP) brings together people who have a common concern or engagement for a topic, and aims to deepen knowledge and expertise around the common issue by facilitating ongoing interaction (30). An underlying assumption of CoP is that learning, and identity are intertwined (29). When it comes to mental health SUs, it concerns the journey from the periphery of the community to full participation in knowledge production and service delivery, and in doing so developing and obtaining a new identity as SU experts. Participation provides the opportunity to renegotiate a stigmatized identity. However, this identity trajectory also applies to researchers and professionals. Through the lens of CoP theory, co-production nurtures a new context where traditional roles in knowledge production are renegotiated. Researchers’ traditional identities as experts and as those in charge, are modified in communities of practice, where purposes, methods and interpretations of results need to be understood through a broadened expertise built on an integration of SUs and professionals’ contributions and perspectives. Applying CoP allows for a theoretical exploration of (knowledge) identity negotiation and involvement in participatory research (29). It may also provide a means of professional reflection on that which is taken for granted, what knowledge is valued and co-production. In the infrastructure of the research program, the concepts of power, knowledge injustice and collaborative learning are supported through a consistent co-production where democratic processes are emphasized, and the knowledge of the various parties is the very core of the activities. In order to have a specific focus on power structures in the program and in the sub-projects, theories such as co-optation (31), participatory spaces (32), and epistemic injustice (33) are applied. They allow for a focus on the power imbalances of public authorities and user groups, and an analysis of how such power relations are affected by SU involvement. Theories on knowledge grounded in lived experience provide the foundation for a discussion of the means and practices of SU knowledge contribution in an EBP (34). Through these theoretical lenses, SU involvement will be analyzed in relation to a renegotiation of power relations, but also in relation to the integration of SUs experiential knowledge in community mental health practice.

## 3. Method

The overarching aim and research activities have been planned for in collaboration with both SU organizations and service providers. SU involvement does not only constitute the study object of this program, but methods of co-production are also at the core of the program design. In the development of the program’s underlying research projects, co-production strategies have been planned for at all phases, involving representatives of SU organizations and mental health services at both local and national levels.

### 3.1. Setting

The setting for the research program is a co-production platform between researchers, welfare actors and The Swedish Partnership for Mental Health (NSPH). The program design, its aim, and the research questions have been developed in close collaboration with NSPH. The national NSPH is an umbrella organization consisting of 13 different mental health organizations whose membership base primarily includes both service users and family members. Its main focus is to drive change on the political level and to develop methods, such as peer support and user focused monitoring, for improving SU involvement in decision making on all levels. In addition to the overall collaboration with the national NSPH, collaborations with the regional NSPH-organizations and other local SU groups are established in the different research projects. This cooperation with the national, regional, and local SU organizations is an important resource for the co-production processes of the program.

The researchers in the program represent five universities in Sweden and are also all affiliated with the Centre for Evidence-based Psychosocial Interventions (CEPI), a national multi-disciplinary research centre with the explicit ambition of integrating SU perspectives in research. CEPI, established in 2007, constitutes an arena for collaboration spanning several national research environments. In addition to national and international research collaborations, CEPI has established close collaborations with national stakeholders, public service providers and SU organizations. CEPI is specialized in research on psychosocial interventions for people with psychiatric disabilities in a community mental health context and has a strong focus on recovery perspectives and SU involvement. The centre has further been leading in developing SU involvement in research, for instance through organizing SU panels for research feedback and evaluation, and by integrating a centre-based SU council. In addition to conducting research, CEPI arranges education and training activities and offers implementation support to welfare actors in the mental health sector. The program will employ CEPI as an infrastructure to build new expertise and strengthened research environments on co-production and SU involvement.

### 3.2. Organization and management

The UserInvolve program has established a program infrastructure for the organization and management of the program (Table 1), consisting of a number of working groups: a strategy group, a program group (management team), a smaller operative group and an international scientific advisory board, Forum UserInvolve and the respective project teams (for the six underlying research projects). These groups, along with the program structure, is key to the management of the research activities and further aims to strengthen co-production, dialogue, and transparency within the program, as well as communicating and disseminating research results.

**Table 1:** Program organization and management.

Groups	Structure					
	Strategy group	The program group (management team)	Operative group (AU)	International scientific advisory board	Forum UserInvolve	Project teams
**Role**	Oversees that the program goals and outputs are achieved and suggests actions if required.	Manages all organizational and scientific processes.	Manages ongoing issues and activities in the program and acts on mandates from the larger program group.	Advisory and supporting role to the management team.	A forum for dissemination and discussion between service providers, service users, and researchers.	Manage each specific research project in the program.
**Purpose**	To provide strategic development advice and provide support, guidance and oversight of the progress and direction of the program.	To plan, develop, coordinate, implement and follow up the activities in the program.	To co-ordinate activities within the program, address general concerns and to continually communicate research results.	To provide advice and support to the management team in scientific issues related to needs, relevance, development, content, focus and aggregated results and synergies.	To offer an online meeting place, where results and experiences from research projects or current initiatives can be presented and discussed in an open atmosphere.	To plan, develop, coordinate, implement and follow up the activities in each specific research project.
**Operator of the meeting/groups**	Program director	Program director	One of the user research coordinators	Responsibility divided between the senior researchers in the program	One of the service user coordinators	Respective project manager
**Participants**	Representatives from NSPH, SALAR, NBHW, Public Health Agency, Swedish Agency for Health Technology Assessment and Assessment of Social Services, City of Stockholm, and Region of Skåne.	The researchers within the program and user- research coordinators from NSPH.	Program director, user- research coordinators, and researchers within the program.	Includes 5–7 senior national and international researchers.	These events are open for all people interested in issues related to service user involvement. So far, a majority of participants are either people with lived experience, or professionals/officials.	Researchers, representatives of local user organizations and of local service providers.
**Frequency**	Four times each year	Four times each year	Biweekly	Depending on need	Six times per year	Regular meetings based on needs.
**Input**	Updated information and status of ongoing activities.	Current topics to discuss related to projects and overall program issues.	Current topics and issues to discuss.	Current topics to discuss related to projects and overall program issues.	Knowledge from current research and initiatives	Current topics to discuss related to the specific project.
**Output**	Guidance in strategic and operational issues.	Strategy, goals, activities, follow-up and decisions.	Developed activities and processes.	Guidance in strategic and operational issues.	New perspectives and insights mediated by service providers and people with service user experience.	Strategy, goals, activities, follow-up and decisions.

An innovative feature of the program management is the integration of a coordinator function for SU involvement. These two coordinators are affiliated with NSPH and are members of the program group. Their main responsibilities are to ensure that the right competence is present in the projects and to make both internal and external communication readily accessible. It is likely that the coordinator role will evolve to reflect the changing needs and demands of the program.

### 3.3. Research activities

The program contains six underlying projects (Table 2). Project 1 is a document study focusing on how SUs knowledge and perspectives are articulated in autonomous arenas for SU mobilization, these being the varied structures that have emerged for SU groups’ engagement in SU involvement activities. Project 2–5 of the program focus on empirical research, with the aim of exploring concrete strategies for sustainable integration of SU involvement in mental health practices. Project 2–5 address different levels of SU involvement: from the individual-, to the organizational- and the systemic level. The objective of these four projects is to develop a better understanding of barriers and success factors in implementing SU involvement strategies in mental health practices. This will be achieved through co-development, execution and evaluation of the following intervention and implementation projects; Shared decision making (SDM), Peer Support (PS), User-focused monitoring (UFM) and system level SU involvement methods. Together, these four studies are expected to contribute to enhanced evidence on methods for strengthened SU participation. Project 6 takes an overarching approach to the knowledge produced in the program, focusing on theory and methodology development relating to co-production in mental health research and practice.

**Table 2:** Overview of the included projects in the program.

Project	Title	Projects objectives	Research aim	Design	Data collection	Data analysis
1	Building a collective knowledge base through popular mobilization	To explore how welfare services can be improved to meet different needs among users with mental illness.	To explore what the needs and knowledge in organized user-movement groups consist of, how knowledge is hierarchized, and how welfare services can be improved based on this knowledge.	Netnographic (online observation and analysis) and ethnographic methodology.	N/ethnographic observations, qualitative interviews, focus group interviews.	Qualitative analysis, both inductive and deductive. Content/thematic analysis, and discourse analysis.
2	Shared Decision-making (SDM) in Coordinated Individual Planning (CIP)	To improve user involvement in care planning in mental health services in Sweden	To identify factors that promote and facilitate a sustainable implementation of SDM in CIP	A multi-case implementation study	Part I. Stakeholder mapping and focus group- and individual interviews will be conducted with stakeholders. Four geographical regions in Sweden will be included.	Qualitative content analysis, both inductive and deductive. Descriptive quantitative analysis.
					Part II. Data collection will be based on interviews and questionnaires with staff and users. The questionnaires will measure SDM and feasibility, usability and acceptability of the intervention.	
3	Peer Support	To contribute to the development of a sustainable peer support intervention for the benefit of service user and mental health services in Sweden	Based on experiences and knowledge of peer support in a Swedish context, co-produce and adapt an international peer support intervention (UPSIDES) and investigate the effectiveness of the intervention delivered in a Swedish mental health service setting	Cross-sectioned mixed methods study	Part I. National survey, focus group- and individual interviews with stakeholders (i.e., peer supporters, supervisors, and managers at a local and regional or municipal level)	Co-produced deductive and inductive content/thematic analysis
				Co-design approach	Part II. Iterative group meeting materials: Fieldnotes, pictures, audio- and video-recordings (zoom)	Co-produced qualitative content analysis
				Parallel, multi-center, randomized controlled trial	Part III. Self-reported questionnaires administered at baseline, post-intervention, and follow-ups (post-intervention). Process evaluation and fidelity measures	Descriptive and inferential statistics
4	User-focused monitoring (UFM)	To improve UFM as a strategy for user involvement in the development of community mental health services	To identify patterns in UFM reports, analyse challenges in the realisation process and investigate the outcomes of UFM.	A mapping study and a multi-case process study, with follow-up.	Part I. Analysis of 136 Swedish UFM reports compiled in a database by NSPH.	Qualitative content analysis, both inductive and deductive.
					Part II. A multi-case process study of five UFM processes. Based on interviews with user monitors and commissioners at the start-up and completion phase.	
					Part III. A follow-up study of the five UFM included in part II. Based on interviews with managers, staff and service users.	
5	User involvement at systemic level	To contribute to the improvement of the action-orientation of system-level involvement structures	To map and analyse system level involvement activities at both local and national levels in Sweden.	Policy analysis and explorative mapping studies at local and national level	Part I: Mapping and analysing systemic user involvement at the national level. Based on policy analysis and key informant interviews with national actors.	Discourse analysis and qualitative content analysis.
					Part II: Mapping and analysing systemic user involvement at the local level. Based on interviews with user representatives, managers and public officials as well as document analysis.	
6	Meta project involving theory and methodology development.	To contribute with synthesized insights and knowledge, and to develop co-production methods in community mental health research and discern theory and general principles for sustainable integration of user involvement in mental health practice.	To explore strategies for integrating co-production in mental health research and practice, examining both hindrances and success-factors.	Instrument development, empirically investigating and developing co-production methods in community mental health research, meta-synthesis.	Part I. Focus group interviews focusing on validity testing of instruments.	Validity and reliability. Meta analysis of data from the projects. Deductive analysis based on theories on knowledge legitimacy, power relations and implementation processes.
					Part II. Co-production activities of the programme will be documented and followed up by focus group interviews at programme- and project level at two occasions.	

#### 3.3.1. Project 1: building a collective knowledge base through popular mobilization

Swedish government official reports (GoR) (5) address the need for evidence-based and competence-enhancing working methods, combined with an increased focus on participation and influence for SUs - in line with the 2030 Sustainable Development Agenda. Concurrently, existing challenges in mental health service organizations and primary care are addressed, highlighting the gap between the ideals of SU participation, and the current situation of individuals with mental illness who are situated as ‘service users’ of welfare state services (2). One issue raised in the GoR, is that of insufficient professional PCC competence in primary care, which would help in recognizing SUs symptoms of mental illness or conforming to SUs needs and/or requested treatment methods. The GoR report further highlights the value and necessity of experiential knowledge in the ongoing development of welfare services for individuals with mental illness, in line with current research (2).

Project 1 is focused on mapping and analyzing activities in autonomous (informal) SU arenas, both online on social media platforms and in physical organizations. Based on both these forms of mobilizations, we will attend to how SUs collectively share experiences and, by way of that, construct a shared knowledge base and political standpoints in the realm of accessible welfare provisions. N/ethnographic methodology will be used, in three different sites, including the collection of observational fieldnotes combined with focus groups and qualitative interviews.

#### 3.3.2. Project 2: implementing shared decision making (SDM) in coordinated individual plans (CIP) through a participatory design

SDM is an evidence-based method recommended in national guidelines since 2012 but has still not been implemented to any greater extent. SDM is a collaborative process, that creates conditions for SUs and professionals to jointly formulate goals and make informed decisions that consider the knowledge of the SU, the provider and available scientific knowledge (35). SDM has been advocated for as an important method for strengthening person-centred care and social practice ((36)) but has received limited attention in community mental health, although studies show that SUs want to be involved in care planning. In Sweden, SDM has been emphasized in the development of coordinated individual care planning (CIP). A CIP is required by law and created when SUs with complex needs require coordinated care from multiple providers. CIPs are required to follow SDM principles in order to promote involvement and collaboration (37), yet this still remains to be realized (37, 38).

The project is designed as a multi-centre study with a participatory approach (39) exploring the implementation of SDM in CIP, with people in community mental health services. A support tool for CIP, including the core concepts of SDM, has previously been co-created with SUs and professionals (7), and will be implemented at four community mental health sites in Sweden. The project includes two parts. The first is a multi-case study that will use a stakeholder mapping approach (40) to examine requirements for successful implementation of SDM in CIP. The second part supports and examines the implementation of SDM in CIP on an individual and organizational level. From an implementation perspective, barriers and success factors for a reinforced SDM in CIP processes are examined, as well as how well SDM has become an integrated part of the CIP work.

#### 3.3.3. Project 3: coproducing the development and evaluation of a peer support intervention to improve social inclusion and recovery in a Swedish mental health service context

Peer support is a service provided by a trained individual with lived experience of mental illness and offers support and hope during treatment to aid in the long-term recovery of mental health SUs (41). A large part of the peer supporter’s role, when working alongside professional providers, is to contribute to increased equality in psychiatric care. Research shows that the peer supporter often influences the climate of the caring environment, which may become more recovery-oriented (42, 43). Despite a growing body of evidence suggesting that peer support has a positive impact on recovery, systematic reviews indicate a need for more rigorous and longitudinal research to understand its essential elements, such as the setting and mode of delivery (44–46). The value of this knowledge acquisition has been recognized in an international peer support project developing a framed peer support intervention, which is being tested in a multinational trial (47, 48). Building on these efforts, this project will work towards co-producing a peer support intervention with stakeholders, one that can be tested in a larger trial.

The project consists of three parts. The first will explore stakeholders’ experiences of the current state of peer support by conducting a national survey and interviews. Secondly, a compilation of the experiential knowledge will be used to subsequently inform, develop, and iterate a scaled-up peer support intervention with inspiration from the international intervention (48, 49) in co-production with stakeholders. The final phase will assess the effectiveness of a peer support intervention on social inclusion through a larger trial in a Swedish mental health service context.

#### 3.3.4. Project 4: do service users’ experiences matter? Examining the practice of user-focused monitoring (UFM) in mental health services

UFM is a systematic and independent method of reviewing care and support services, with the entire process performed by people with lived experience of mental illness (50, 51). There is today broad consensus on the importance of including SU perspectives in quality development both in Sweden and internationally (20, 52). However, knowledge of the outcomes of involvement methods remains limited (53, 54). Specifically, research on user involvement at the organizational level (53) and studies on service monitoring and evaluation (54) are lacking. The project addresses this knowledge gap by examining UFM as a strategy for SU involvement and quality development in mental health practice.

The project consists of three parts. The first is focused on mapping and analysing Swedish UFM reports, compiled in a database by NSPH. The second part is a multi-case process study of five UFM processes. The third part is a follow-up study focused on the outcomes and significance of UFM.

#### 3.3.5. Project 5: user involvement at a systemic level

Since the 1990s, Swedish mental health SU organizations have participated in government projects and commissions of inquiry (16) These organizations currently have a significant role in democratizing and developing the quality of the welfare system. However, several reports have highlighted how SU involvement at the systemic level is lacking, especially within the mental health sector. Within the SU movement, the lack of action-oriented SU involvement methods, such as user councils, at the systemic level have regularly been discussed. This project addresses this problem, by analyzing system level involvement activities with a specific focus on their contribution to actual change in welfare organizations.

The project consists of two parts. The first is focused on systemic SU involvement at the national level, through mapping and analysing policy documents, as well as national actor’s initiatives regarding SU involvement. The second part of the project takes an explorative approach to map and analyse activities at a systemic local level.

#### 3.3.6. Project 6: meta project involving theory and methodology development

This project is divided into three parts and takes an overarching approach to the knowledge generated in the program, in order to develop theory and methodology in relation to SU involvement in research and in mental health practices. The first part is focused on the development and validation of questionnaires and interview guides that will be applied to develop and evaluate the co-production activities within the program. Part two focuses on empirically investigating and developing co-production methods in mental health research. In this part, our focus will be to explore strategies for integrating co-production in mental health research, examining both barriers and success factors. In the third part we aim to draw on insights generated in the program at-large in order to develop theory that can be applicable to a range of user involvement practices.

### 3.4. Co-production in the six projects

Our co-production ambition is reflected at both the project and program level. Program level co-production arenas, such as the strategy group, the user research coordinators and Forum UserInvolve are described in Table 1. Co-production is further integrated in the research process of each project, but the format and method for involving co-production partners are based on the needs and focus of each research project. For example, in project 1, a user committee comprising individuals with personal experiences of social and mental health services, serve as a reference group in ongoing analysis of data. In project two, a close collaboration with an SU driven Recovery College (55) has been established. The collaboration is about co-creating forms for a long-term implementation of training for professionals in shared decision-making in care planning. Project three, which addresses peer support, will emphasize the co-production of planning, performance, and analysis of both qualitative and quantitative research with the peer support practice. For project four and five we have established reference groups for the projects. These groups involve representatives of the SU movement and of the mental health service system with knowledge and experience of the specific method of SU involvement that is investigated in the respective projects. These groups provide input throughout the research process, by contributing to formulating and prioritizing research needs, by providing input on data collection and analytical procedures, and by contributing to the dissemination of results. Co-production at both the program and project levels, emphasizes flexibility in methods and formats. These include the use of co-creation and co-design methods for research, and the importance of sharing the outcomes of co-production efforts. This approach fosters a more inclusive, multidisciplinary, and impactful approach to knowledge generation and seeks to ensure that the perspectives and contributions of various stakeholders are valued and integrated into the program.

### 3.5. Time plan and execution

**Figure 1:**
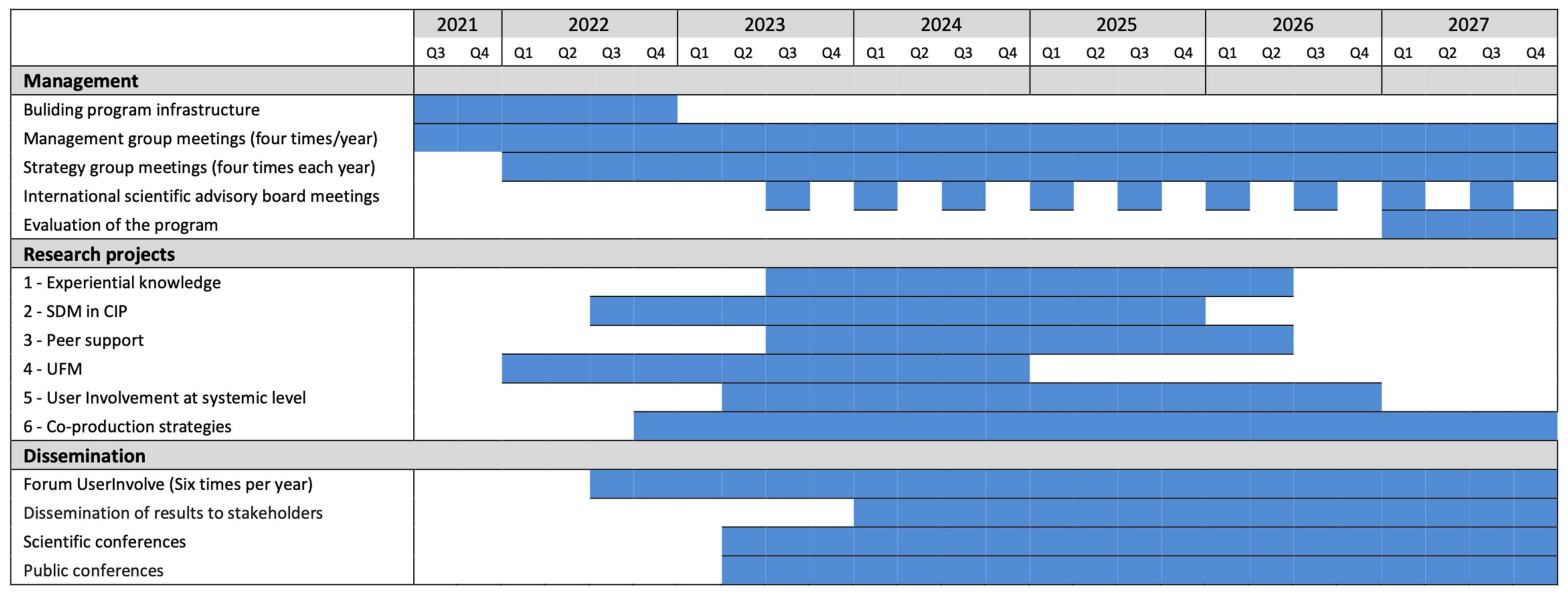
Program timeplan.

**Figure 2:**
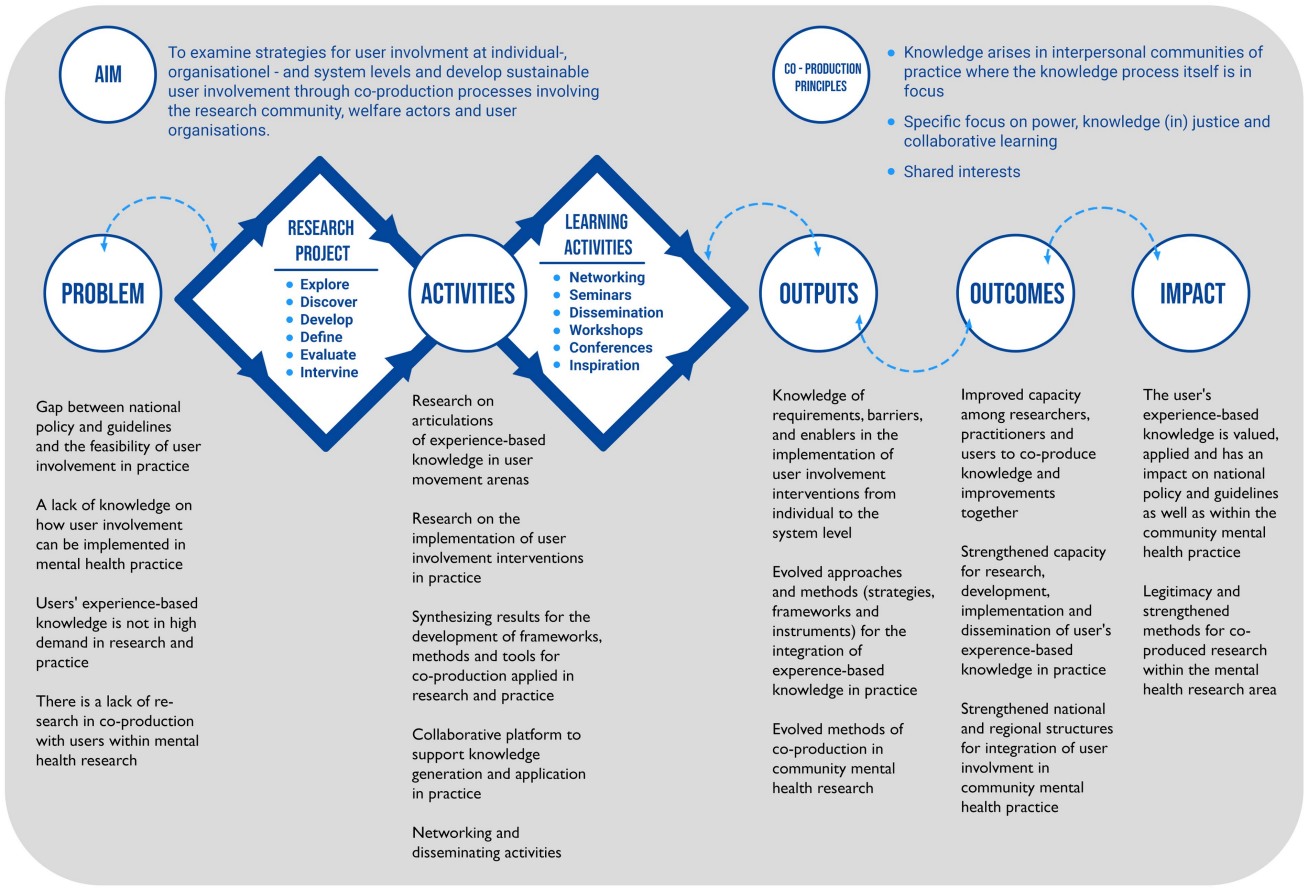
Logic model for the UserInvolve program.

The UserInvolve program formally started in January 2022 through the formation of a program group, the development of the program’s infrastructure and an action plan for running the program until the end of 2027. The program is proceeding according to the time plan presented in Figure 1. The program began with the development of a logic model, which is a common approach for planning and evaluating projects and/or programs (56) in order to achieve their overall goals (see Figure 2). The logic model will be used to link the problem (needs) to the activities, outputs, outcomes and impacts and through this the model will support planning, monitoring and evaluation of the program. Thus, a combination of activities, both research project activities and learning and dissemination activities, have been identified as necessary to achieve the outputs (measurable results) required to accomplish the desired change (outcomes) and the impact (the ultimate and strategic influence we want to achieve) within the scope of the program. Six research projects will be carried out and these projects have different schedules as presented in the time plan (Figure 1).

### 3.6. Dissemination

A number of activities will be focused on establishing a communication and dissemination platform for the research program. The co-produced ambition of the program means that we have the goal of creating an ongoing dialogue, where research results are continuously reported, feedback is received, and new research ideas are discussed. The communication and dissemination will be tailored to the needs and preferences of different audiences to maximizes the impact of the research. Thus, we will use varied dissemination activities, co-produced with our stakeholders in the program and projects, for potentially achieving greater impact than exclusively researcher-driven dissemination activities.

The main arena for communication and dissemination will be the digital seminar format “Forum UserInvolve” (see Table 1). This seminar is moderated by one of the user involvement co-ordinators and focuses on the reporting of current research results concerning user involvement issues. The involved researchers will present current studies at these seminars, and other researchers will also be invited to present on themes relevant to the program. Presentations are followed by a joint dialogue among the seminar attendees on the theme in focus. These seminars target SUs, practitioners, researchers, and policymakers as its audience, aiming to contribute to a dialogue among actors representing relevant perspectives.

The involved researchers will present at scientific peer-reviewed conferences (e.g., Refocus on Recovery, ENMESH and European Conference for Social Work) as well as public conferences (e.g., arranged by co-production partners). To enable a broader public reach, social media activities will also be integrated in the program and its strategies for knowledge dissemination. A UserInvolve Facebook page will serve as a forum for disseminating the research results to a broader public, but also as a communication platform for the network on SU involvement issues. A podcast produced by NSPH will serve as an additional forum for broader dissemination of the research results.

Collectively, these strategies aim to contribute to the formation of a network on SU involvement issues, assembled in collaboration with SU organizations and mental health representatives, thereby strengthening translation of the knowledge generated in the program into sustainable practice.

## 4. Discussion

At a general level, the UserInvolve-program can contribute to the quality development of mental health services by examining how the SUs opinions, values and knowledge can be integrated into practice. Motives for increased SU involvement are related to democratic participation, redistribution of power, and to service system adjustments (57). SU involvement can create more ethical mental health systems from a human rights perspective but is additionally related to quality development in services since SU involvement can contribute to more efficient services (20, 57). The legitimacy of the mental health system can further be strengthened by the SUs attaining greater transparency and insight into policies and decisions (57). Moreover, SU involvement can itself be seen as a form of health promotion as it contributes to empowerment, personal recovery (58) and is a crucial aspect of democratic practices (59).

UserInvolve is focused on development, change and implementation work. It supports the implementation of four specific interventions for SU involvement and through these examines how SU involvement can be integrated in mental health practice. Our ambition is thereby to generate knowledge of more general components that are relevant to a range of involvement strategies. The involvement interventions examined may be relevant to further employ in both health- and social care services. The program will contribute with knowledge of how user involvement is enacted in these organizational contexts, but also of specific challenges associated with co-production in these sectors. A multitude of SU involvement initiatives are currently emerging in Sweden, but many of these assume the form of short-term projects. It is therefore relevant to examine and develop sustainable implementation. We further regard the co-production processes in the program as crucial for accomplishing such transformative changes in mental health practice. In addition, the program makes a contribution by studying the effects of SU involvement interventions. Evidence of such effects have been called for in prior studies, and the UserInvolve-program examines individual level outcomes as well as outcomes at an organizational level.

The co-production structures of the program are built on ambitions to generate an evolved understanding of how SU involvement can be enacted and what significance it has for individual SUs and for SUs as a group in society. SU movement and welfare actors participating in UserInvolve have a key role in this, by contributing with their knowledge perspectives to the exploration of interventions, and of barriers and facilitating factors to sustainable implementation. Through our co-production approach, we further have the ambition to develop networks for collaborative knowledge processes or communities of practice, that extend beyond the program years. The program will further contribute to co-production theory and methodology development, by developing instruments to follow up co-production processes and impact and by discerning more general principles regarding the sustainable integration of SU involvement in mental health systems.

In conclusion, the program has both conceptual and instrumental benefits for SU involvement policy and practice. Conceptually, the program has the ambition of contributing to general awareness, literacy and readiness for SU involvement among providers and enabling SUs to be empowered and supported as they attempt to participate in their care. The contributions of the program that regard co-production theory and methodology can further be relevant to the research community, but also to other actors that have ambitions to integrate a co-production approach. Instrumentally, the program intends to develop SU involvement strategies and improve implementation processes. This would contribute to an extended and refined repertoire of strategies for mental health research and practice. The co-production structures of the program further involve central government authorities and may thereby contribute to a policy-level impact based on their experiences and the knowledge generated in the program.

## Ethics statement

The ethical considerations in these six underlying projects are based on their specific methodological procedures. These projects have, or will, all undergo individual ethical review.

## Author contributions

UM: Conceptualization, Data curation, Formal analysis, Funding acquisition, Investigation, Methodology, Project administration, Resources, Supervision, Writing – original draft, Writing – review & editing. HN: Writing – review & editing, Conceptualization, Data curation, Formal analysis, Funding acquisition, Investigation, Methodology, Project administration, Writing – original draft. U-KS: Conceptualization, Data curation, Formal analysis, Funding acquisition, Investigation, Methodology, Project administration, Resources, Supervision, Writing – original draft, Writing – review & editing. DR: Conceptualization, Data curation, Formal analysis, Funding acquisition, Investigation, Methodology, Project administration, Resources, Supervision, Writing – original draft, Writing – review & editing. UB: Conceptualization, Data curation, Formal analysis, Funding acquisition, Investigation, Methodology, Project administration, Resources, Supervision, Writing – original draft, Writing – review & editing. AG: Conceptualization, Data curation, Formal analysis, Funding acquisition, Investigation, Methodology, Resources, Writing – review & editing. MJ: Conceptualization, Data curation, Formal analysis, Funding acquisition, Investigation, Methodology, Resources, Writing – review & editing. EA: Conceptualization, Data curation, Formal analysis, Funding acquisition, Investigation, Methodology, Project administration, Resources, Supervision, Writing – original draft, Writing – review & editing. KG: Conceptualization, Data curation, Formal analysis, Funding acquisition, Investigation, Methodology, Project administration, Resources, Writing – original draft, Writing – review & editing. PE: Conceptualization, Data curation, Formal analysis, Investigation, Methodology, Resources, Writing – original draft, Writing – review & editing. FN: Conceptualization, Data curation, Formal analysis, Investigation, Methodology, Resources, Writing – original draft, Writing – review & editing. SL: Conceptualization, Data curation, Formal analysis, Investigation, Methodology, Resources, Writing – original draft, Writing – review & editing. PS: Conceptualization, Data curation, Formal analysis, Funding acquisition, Investigation, Methodology, Project administration, Resources, Supervision, Visualization, Writing – original draft, Writing – review & editing.
